# Supplementary material for: Neuroanatomical Correlates of Social Intelligence Measured by the Guilford Test
Source: Brain Topogr. 2021 Apr 18;34(3):337–47. doi: 10.1007/s10548-021-00837-1 (PMC8099826; doi:10.1007/s10548-021-00837-1)
Supplement: Supplementary file 1 — Supplementary file1 (DOCX 637 kb) [file 10548_2021_837_MOESM1_ESM.docx]

**Supplementary Data:**

Neuroanatomical correlates of social intelligence measured by the Guilford test

Myznikov A^1^., Zheltyakova M^1^., Korotkov A^1^., Kireev M^1,4^., Masharipov R^1^., Jagmurov O.Dz.^1^, Habel U^2,3^., Votinov M^1,2^

**Supplementary Figure 1. Results of correlational analysis for sum of tests using z-scores (p<0.001, uncorrected, minimal cluster size – 10)**

**
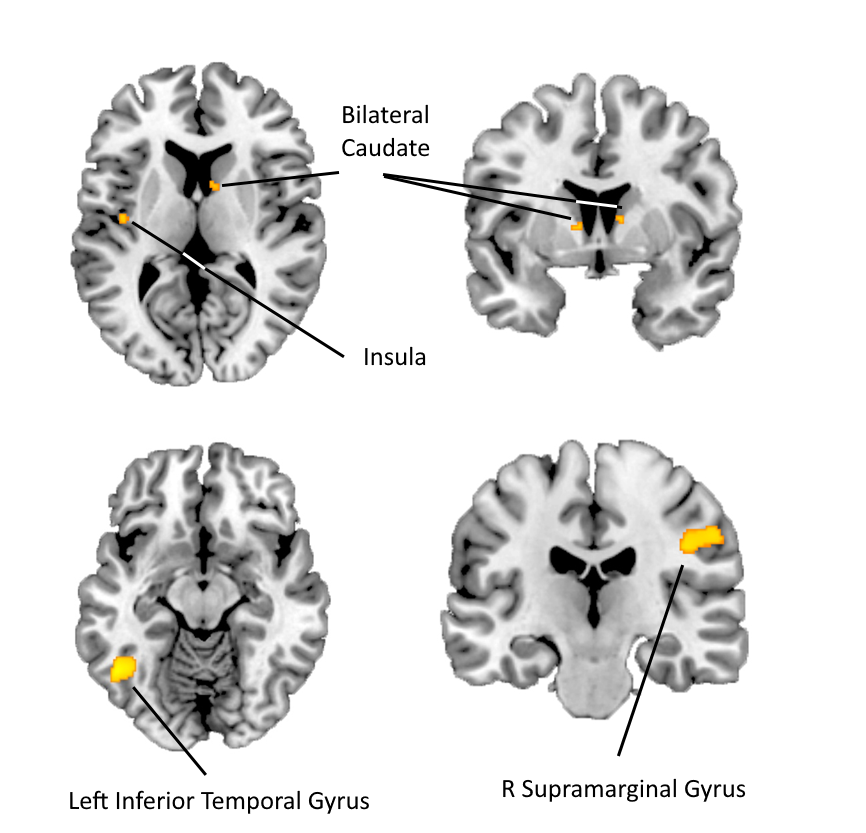
**

**Supplementary Table 1. Results of correlational analysis for sum of tests using z-scores (p<0.001, uncorrected, minimal cluster size – 10)**

| **Region (L - left, R - right)** | **Cluster size (k)** | **T score** | **MNI coordinates** | | |
| --- | --- | --- | --- | --- | --- |
|  |  |  | **x** | **y** | **z** |
| L Inferior Temporal Gyrus  L Fusiform Gyrus | 459 | 5.34 | -48 | -57 | -12 |
| R Supramarginal Gyrus | 445 | 4.47 | 54 | -25.5 | 37.5 |
| L Superior Parietal Lobule | 32 | 3.79 | -40.5 | -52.5 | 58.5 |
| L Middle Temporal Gyrus  L Inferior Occipital Gyrus | 144 | 3.75 | -37.5 | -66 | 3 |
| R Angular Gyrus | 37 | 3.65 | 39 | -66 | 52.5 |
| L Precuneus | 25 | 3.64 | -3 | -51 | 58.5 |
| R Superior Parietal Lobule | 10 | 3.50 | 25.5 | -42 | 46.5 |
| L Posterior Insula | 13 | 3.48 | -40.5 | -12 | 10.5 |
| L Caudate | 35 | 3.48 | -7.5 | -1.5 | 0 |
| R Caudate | 10 | 3.39 | 9 | 6 | 9 |

**Supplementary Figure 2. Results of correlational analysis for 4th subtest z-scores (p<0.001, uncorrected, minimal cluster size – 10)**

**
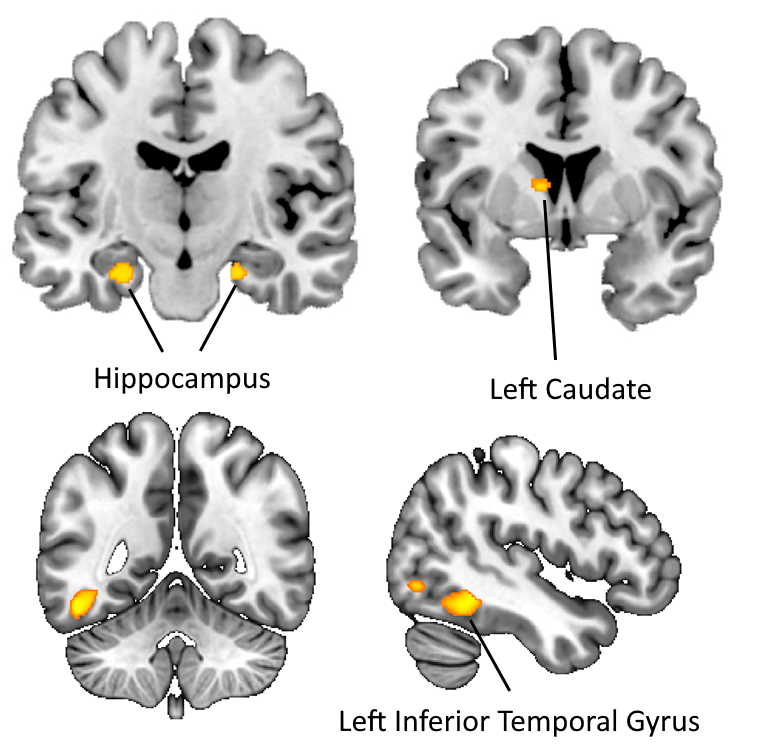
**

**Supplementary Table 2. Results of correlational analysis for 4th subtest z-scores (p<0.001, uncorrected, minimal cluster size – 10)**

| **Location** | **Cluster size (k)** | **T score** | **MNI coordinates** | | |
| --- | --- | --- | --- | --- | --- |
|  |  |  | **x** | **y** | **z** |
| L Inferior Temporal Gyrus  L Fusiform Gyrus | 494 | 4.8 | -40.5 | -45 | -12 |
| L Inferior Occipital Gyrus | 133 | 4.29 | -42 | -72 | -6 |
| L Superior Frontal Gyrus | 34 | 4.04 | -12 | 49.5 | 24 |
| L Hippocampus | 126 | 4.01 | -25.5 | -12 | -24 |
| R Hippocampus | 66 | 3.86 | 21 | -13.5 | -22.5 |
| R Inferior Occipital Gyrus | 40 | 3.77 | 39 | -78 | -7.5 |
| R Postcentral Gyrus  R Supramarginal Gyrus | 22 | 3.70 | 43.5 | -18 | 34.5 |
| R Postcentral Gyrus | 22 | 3.6 | 19.5 | -40.5 | 75 |
| L Anterior Orbital Gyrus | 10 | 3.55 | -28.5 | 54 | -9 |
| L Caudate | 28 | 3.54 | -10.5 | 3 | 9 |
| R Middle Frontal Gyrus | 13 | 3.52 | 25.5 | 22.5 | 36 |
